# MYD88, NFKB1, and IL6 transcripts overexpression are associated with poor outcomes and short survival in neonatal sepsis

**DOI:** 10.1038/s41598-021-92912-7

**Published:** 2021-06-28

**Authors:** Nouran B. AbdAllah, Eman A. Toraih, Essam Al Ageeli, Hala Elhagrasy, Nawal S. Gouda, Manal S. Fawzy, Ghada M. Helal

**Affiliations:** 1 Department of Pediatrics, Faculty of Medicine, Suez Canal University, Ismailia, Egypt; 2 Department of Surgery, School of Medicine, Tulane University, New Orleans, LA USA; 3 Genetics Unit, Department of Histology and Cell Biology, Faculty of Medicine, Suez Canal University, Ismailia, Egypt; 4 Department of Clinical Biochemistry (Medical Genetics), Faculty of Medicine, Jazan University, Jazan, Saudi Arabia; 5 Department of Medical Microbiology and Immunology, Faculty of Medicine, Mansoura University, Mansoura, Egypt; 6 Department of Medical Biochemistry and Molecular Biology, Faculty of Medicine, Suez Canal University, Ismailia, Egypt; 7 Department of Biochemistry, Faculty of Medicine, Northern Border University, Arar, Kingdom of Saudi Arabia; 8 Department of Medical Biochemistry, Faculty of Medicine, Mansoura University, Mansoura, Egypt

**Keywords:** Sepsis, Biotechnology, Genetics, Immunology, Molecular biology, Biomarkers, Diseases, Molecular medicine, Pathogenesis

## Abstract

Toll-like receptor (TLR) family signature has been implicated in sepsis etiopathology. We aimed to evaluate the genetic profile of TLR pathway-related key genes; the myeloid differentiation protein 88 (*MYD88*), IL1 receptor-associated kinase 1 (*IRAK1*), the nuclear factor kappa-B1 (*NFKB1*), and interleukin 6 (*IL6*) in the blood of neonates with sepsis at the time of admission and post-treatment for the available paired-samples. This case–control study included 124 infants with sepsis admitted to the neonatal intensive care unit and 17 controls. The relative gene expressions were quantified by TaqMan Real-Time qPCR and correlated to the clinic-laboratory data. *MYD88*, *NFKB1*, and *IL6* relative expressions were significantly higher in sepsis cases than controls. Higher levels of *MYD88* and *IL6* were found in male neonates and contributed to the sex-based separation of the cases by the principal component analysis. ROC analysis revealed MYD88 and NFKB1 transcripts to be good biomarkers for sepsis. Furthermore, patients with high circulatory *MYD88* levels were associated with poor survival, as revealed by Kaplan–Meier curves analysis. MYD88, NFKB1, and IL6 transcripts showed association with different poor-outcome manifestations. Clustering analysis split the patient cohort into three distinct groups according to their transcriptomic signature and CRP levels. In conclusion, the study TLR pathway-related transcripts have a gender-specific signature, diagnostic, and prognostic clinical utility in neonatal sepsis.

## Introduction

Neonatal sepsis is a case of bacteremia presented clinically with a dysregulated response to infection in the first four weeks of life^1^. As a leading cause of neonatal morbidity and mortality, it remains a significant global health challenge, especially in low-income countries with low health care resources^2,3^. Across all age groups, the highest sepsis incidence is found in neonates, with an estimated 3 million cases globally (22/1000 live births) and about 11–19% death rate with long-term neurological sequelae^4^.

Early clinical diagnosis is still a high suspicion due to a lack of specific early diagnostic tools or specific signs and symptoms. Also, blood culture, as the gold standard of lab diagnosis, has certain limitations, which collectively delay early identification^5^. Routinely, 7–13% of neonates are treated for possible sepsis empirically because of these diagnostic limitations. Molecular signatures linked with a better understanding of the immature neonatal immune system response to early infection would provide an opportunity to develop critically needed biomarkers^6,7^.

**Figure 1:**
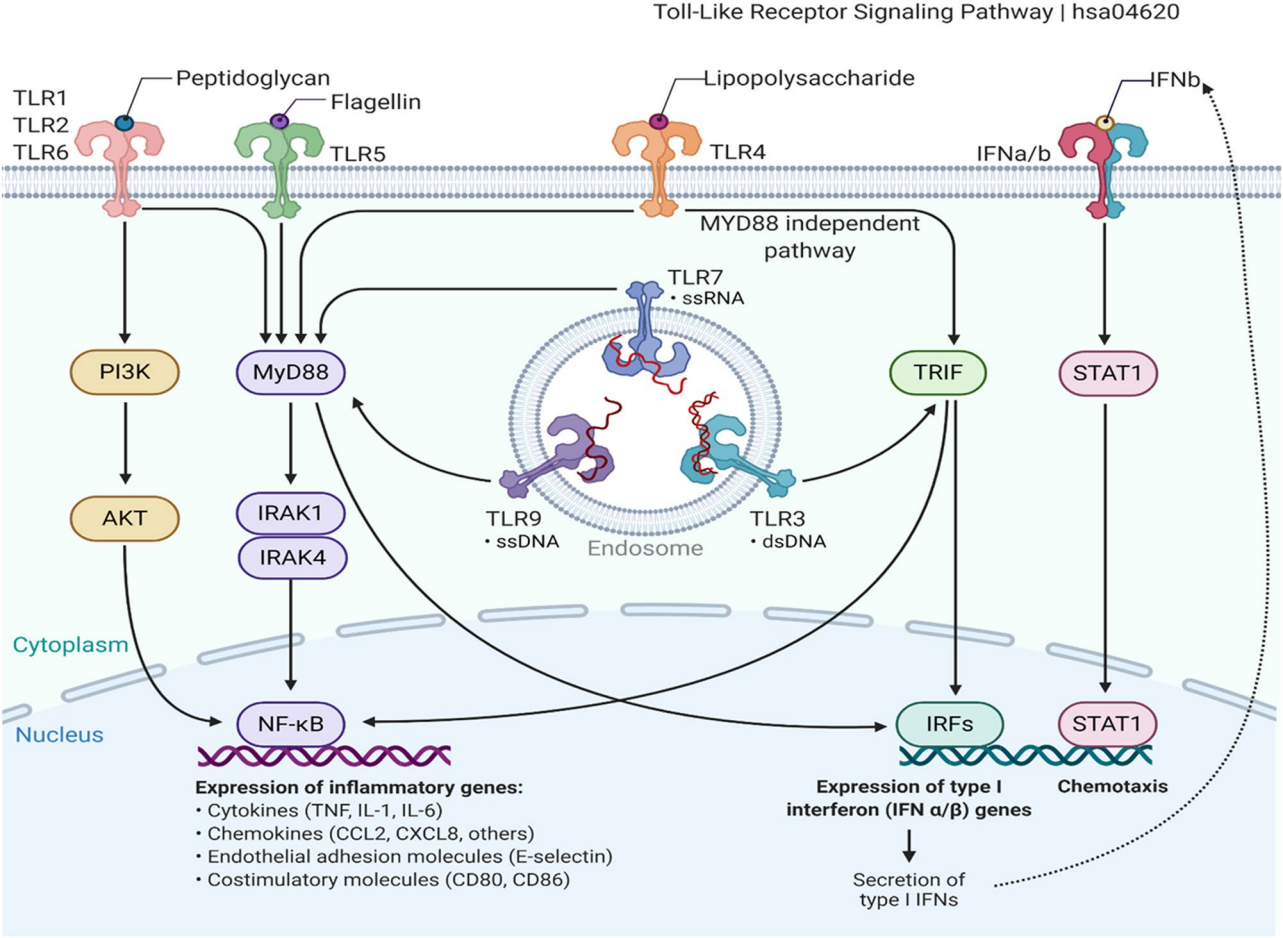
Toll-like receptor (TLR) signaling pathway. On exposure to pathogens secreting pathogen-associated molecular patterns (PAMPs), their recognition is initiated via pattern recognition receptors, including several Toll-like receptors (TLRs). For example, lipopolysaccharides on gram-negative bacteria activate TLR4, lipoteichoic acid of gram-positive bacteria activates TLR2, while TLR3 recognizes viral PAMPs, generating innate immune responses via the MyD88-dependent pathway that leads to the production of pro-inflammatory cytokines with activation of nuclear factor kappa B (NFKB) and the downstream gene targets^9,10^. The MyD88-independent pathway associated with the induction of type I interferon (IFN) and IFN-inducible genes [Data source: KEGG pathway, hsa04620 and created by Biorender.com].

Our in silico and functional enrichment analyses for differentially expressed genes in neonatal sepsis (detailed in the methods section) have identified "Toll-like receptor (TLR)" and "nuclear factor kappa B (NFκB)" signaling pathways as top deregulated pathways in neonatal sepsis. Activation of TLRs, as shown in Fig. 1, recruit several downstream adaptor molecules, including the major player "myeloid differentiation protein 88 (MyD88)", which contributes to signaling amplification through interleukin-receptor-associated kinase (IRAK) family proteins. Convergence of IRAK at the NFκB induces the transcription of interleukin 6 (IL6) that counteracts the threat imposed by the invaders and participates in stimulating other immune components^5,8–10^. Identifying the molecular profile of these genetic signatures may provide novel diagnostic/prognostic clinical utility and/or predict neonatal outcomes. In this sense, the present work aimed to evaluate the genetic profile of TLR pathway-related key players (i.e. MYD88, IRAK1, NFKB, and IL6) in the blood of neonates at the time of admission and after antibiotic treatment (for three days) to evaluate the change in gene expression signature and to correlate the expression levels with the available clinic-laboratory findings.

## Results

### Baseline characteristics of the study population

The study included 124 sepsis infants (81 males and 43 females) and 17 healthy controls (12 males and 5 females) (*p* = 0.78). Both study groups were matched for baseline demographic parameters such as age at sampling (2 ± 1.1 days versus 3.3 ± 3.9, *p* = 0.17), gestational age (37.1 ± 1.9 weeks versus 36 ± 3.6 weeks, p = 0.22), and birth weight (2.6 × 10–3 ± 0.4 × 10–3 g versus 2.4 × 10–3 ± 0.7 × 10–3 g, *p* = 0.16) in cases and controls, respectively. Of the sepsis cases, 93 were early-onset (onset of sepsis features within 72 h of life), while 31 neonates presented with late-onset sepsis (onset of sepsis features after 72 h of life). Comparison between male and female patients is presented in Table 1. A higher frequency of premature rupture of membranes was observed in male newborns (42% vs. 23.3%, *p* = 0.049). In male neonates, they were more likely to be presented with hemodynamic instability (40.7% vs 16.3%, *p* = 0.008), lethargy (53.1% vs 27.9%, *p* = 0.008), and poor feeding (67.9% vs 32.6%, *p* < 0.001). A higher prevalence of congenital pneumonia was reported in male patients (25.9% vs. 9.3%, *p* = 0.034). Also, they were nearly three times more likely to develop complications (OR = 2.71, 95%CI: 1.11–6.6, *p* = 0.025), respiratory failure (*p* = 0.027), and multiple organ failure (*p* = 0.033). Compared to female septic cases, males were three times more at risk of mortality (OR = 2.87, 95% CI: 1.13–7.26, *p* = 0.024) (Table 1).

**Table 1:** Baseline characteristics of the neonatal sepsis.

Characteristics	Levels	Total sepsis	Females	Males	*p*-value	OR (95%CI)
**Demographics**						
Age, days	Median (IQR)	1 (0–3)	2 (0.8–3)	1 (0–3)	0.38	
Gestational age at birth, weeks	Median (IQR)	30 (30–34)	30 (29–30)	32 (30–37.5)	0.94	
Mode of delivery	Vaginal	59 (47.6)	18 (41.9)	41 (50.6)	0.45	Reference
	Cesarean	65 (52.4)	25 (58.1)	40 (49.4)		0.7 (0.33–1.48)
Weight at birth (Kg)	Median (IQR)	1.9 (1.4–2.7)	1.7 (1.1–2.7)	2 (1.4–2.7)	0.20	
	LBWB	12 (9.7)	4 (9.3)	8 (9.9)	0.91	1.07 (0.3–3.77)
Maternal antibiotic	Amoxycillin	17 (13.7)	4 (9.3)	13 (16)	0.41	1.86 (0.57–6.11)
Risk factors	Positive	89 (71.8)	27 (62.8)	62 (76.5)	0.14	1.93 (0.87–4.32)
Maternal factors	PROM	44 (35.5)	10 (23.3)	34 (42)	**0.049**	2.39 (1.04–5.5)
	Maternal diabetes	4 (3.2)	2 (4.7)	2 (2.5)	0.60	0.52 (0.07–3.82)
	Maternal anemia	12 (9.7)	6 (14)	6 (7.4)	0.33	0.49 (0.15–1.63)
	Maternal hypertension	4 (3.2)	0 (0)	4 (4.9)	0.29	0.64 (0.56–0.73)
	Pre-eclampsia	5 (4)	2 (4.7)	3 (3.7)	0.79	0.79 (0.13–4.91)
	Triple I	24 (19.4)	8 (18.6)	16 (19.8)	0.87	1.08 (0.42–2.77)
Neonatal factors	Preterm	53 (42.7)	16 (37.2)	37 (45.7)	0.44	1.42 (0.67–3.03)
	IUGR	6 (4.8)	0 (0)	6 (7.4)	0.09	0.64 (0.55–0.73)
	Total parental nutrition	41 (33.1)	13 (30.2)	28 (34.6)	0.69	1.22 (0.55–2.7)
	Umbilical venous catheter	24 (19.4)	8 (18.6)	16 (19.8)	0.87	1.08 (0.42–2.77)
**Presentation**						
Onset	Early Onset Sepsis	31 (25)	14 (32.6)	17 (21)	0.19	Reference
	Late Onset Sepsis	93 (75)	29 (67.4)	64 (79)		1.82 (0.79–4.18)
Features	TTN	15 (12.1)	2 (4.7)	13 (16)	0.08	3.92 (0.84–18.25)
	Temperature instability	31 (25)	8 (18.6)	23 (28.4)	0.28	1.73 (0.7–4.3)
	Hemodynamic instability	40 (32.3)	7 (16.3)	33 (40.7)	**0.008**	3.54 (1.4–8.9)
	Hypoxia	89 (71.8)	28 (65.1)	61 (75.3)	0.29	1.63 (0.73–3.66)
	Lethargy	55 (44.4)	12 (27.9)	43 (53.1)	**0.008**	2.92 (1.32–6.48)
	Hypoglycaemia	21 (16.9)	9 (20.9)	12 (14.8)	0.45	0.66 (0.25–1.71)
	Poor feeding	69 (55.6)	14 (32.6)	55 (67.9)	** < 0.001**	4.38 (1.99–9.66)
	Seizures	8 (6.5)	2 (4.7)	6 (7.4)	0.71	1.64 (0.32–8.5)
	Oliguria	22 (17.7)	9 (20.9)	13 (16)	0.62	0.72 (0.28–1.86)
	RDS	47 (37.9)	18 (41.9)	29 (35.8)	0.56	0.77 (0.36–1.65)
	Congenital pneumonia	25 (20.2)	4 (9.3)	21 (25.9)	**0.034**	3.41 (1.09–10.7)
	Acquired pneumonia	15 (12.1)	10 (23.3)	5 (6.2)	**0.009**	0.22 (0.07–0.68)
Comorbidities	Congenital heart disease	8 (6.5)	2 (4.7)	6 (7.4)	0.71	1.64 (0.32–8.5)
	Jejunal atresia	3 (2.4)	3 (7)	0 (0)	**0.040**	0.33 (0.26–0.43)
	Inguinal hernia	5 (4)	0 (0)	5 (6.2)	0.16	0.64 (0.56–0.73)
Laboratory data	Total WBC count × 10^3^	7.0 (4.5–12.0)	6.5 (4.8–11.0)	7.0 (4.25–12.8)	**0.029**	
	Immature/Total WBCs count	0.2 (0.2–0.3)	0.2 (0.2–0.3)	0.2 (0.2–0.3)	0.05	
	Platelets count × 10^3^	121 (100–173)	168 (106.3–267.8)	120 (95–150)	**0.024**	
	CRP (mg/L)	5.4 (1–11.7)	5.1 (0.9–11.9)	5.4 (1.0–10.4)	**0.004**	
	Hb (g/dL)	12 (11–13)	12.3 (10.6–13.6)	12 (10.7–13)	0.99	
	Na (mEq/L)	142 (136–146)	141.5 (137–147.3)	142 (136–145)	0.34	
	K (mmol/L)	4.2 (4–4.6)	4.3 (4–4.4)	4 (4–4.9)	0.58	
	Ca (mg/dL)	8.7 (8.3–9)	9 (8.9–11.6)	8.6 (8.2–9)	0.07	
	pH	7.3 (7.2–7.3)	7.3 (7.2–7.4)	7.2 (7.2–7.3)	0.16	
	PCO2 mmHg	55 (41–60)	55.5 (52.8–65)	55 (40.2–59.5)	0.50	
	PO2 mmHg	65 (40–78)	69 (56.3–79.8)	65 (39.5–74)	0.09	
	HCO3 mEq/L	19 (15–22)	21 (18.5–23.3)	18 (15–21.4)	0.85	
Culture data	Culture	91 (73.4)	36 (83.7)	55 (67.9)	0.08	0.41 (0.16–1.05)
	Positive results	51 (41.1)	17 (39.5)	34 (42)	0.84	1.11 (0.52–2.35)
Type of organism	E. coli	13 (25.5)	6 (35.3)	7 (20.6)	0.16	Reference
	Klebsiella	15 (29.4)	4 (23.5)	11 (32.4)		2.35 (0.48–11.4)
	Staph aureus	15 (29.4)	3 (17.6)	12 (35.3)		3.42 (0.64–18.2)
	Streptococcus	6 (11.8)	4 (23.5)	2 (5.9)		0.42 (0.05–3.22)
	Pseudomonas	2 (3.9)	0 (0)	2 (5.9)		4.33 (0.17–107)
**Management**						
Antibiotics	B-lactamse combinations + third generation cephalosporin	73 (59.8)	25 (61)	48 (59.3)	0.96	
	Penicillin + aminoglycosides	16 (13.1)	6 (14.6)	10 (12.3)		
	Vancomycin + Carbapenem	14 (11.5)	5 (12.2)	9 (11.1)		
	Fourth generation cephalosporin	8 (6.6)	2 (4.9)	6 (7.4)		
	Others	11 (9)	3 (7.3)	8 (9.9)		
Ventilation	Negative	33 (26.6)	15 (34.9)	18 (22.2)	0.12	Reference
	O2 therapy	42 (33.9)	16 (37.2)	26 (32.1)		0.88 (0.37–2.11)
	Ventilation	49 (39.5)	12 (27.9)	37 (45.7)		2.56 (0.99–6.61)
Surfactant therapy	Positive	21 (16.9)	6 (14)	15 (18.5)	0.62	1.4 (0.5–3.92)
Outcomes						
Length of stay in hospital		14 (8–15)	14 (8–14)	12 (8.5–15)	0.98	
Complication	Positive	39 (31.5)	8 (18.6)	31 (38.3)	**0.025**	2.71 (1.11–6.6)
Type of complications	Pulmonary hemorrhage	6 (4.8)	2 (4.7)	4 (4.9)	0.94	1.06 (0.19–6.06)
	VAP	10 (8.1)	2 (4.7)	8 (9.9)	0.49	2.25 (0.46–11.08)
	Air leak syndrome	10 (8.1)	5 (11.6)	5 (6.2)	0.31	0.5 (0.14–1.83)
	Respiratory failure	9 (7.3)	0 (0)	9 (11.1)	**0.027**	1.60 (1.39–1.84)
	Organ failure	8 (6.5)	0 (0)	8 (9.9)	**0.033**	1.59 (1.38–1.83)
	Necrotizing enterocolitis	4 (3.2)	0 (0)	4 (4.9)	0.29	1.56 (1.36–1.78)
Survival	Survived	88 (71)	36 (83.7)	52 (64.2)	**0.024**	2.87 (1.13–7.26)
	Died	36 (29)	7 (16.3)	29 (35.8)		
Time to death, days		14 (8–15)	14 (8–14)	12 (8.5–15)	0.58	

Data are presented as number and percentage or median and interquartile range (IQR). Tow-sided Chi-square or Mann–Whitney U tests were used. Bold values indicate significance at *p*-value < 0.05. Abbreviations; Triple I: Intrauterine inflammation, infection, or both (a new concept of chorioamnionitis), IUGR: Intrauterine growth retardation. LBWB: Low birth-weight baby. PROM: Premature rupture of membrane. TTN: Transient tachypnea of newborns, RDS: Respiratory distress syndrome, WBC: white blood cells, Hb: hemoglobin, CRP: C-reactive protein, E. coli: Escherichia coli, VAP: Ventilation-association pneumonia, other antibiotics include quinolones or nitroimidazole, or other combinations.

### Bioinformatic selection of Toll-like receptor pathway

The transcriptomic analysis of 324 differentially expressed genes (DEGs), including 305 upregulated and 19 downregulated genes in preterm infants during late-onset sepsis, is shown in Figure S1. Functional enrichment analysis of DEGs identified *TLR* signaling pathway (hsa04620 | hits = 13 out of 104 | FDR = 7.93e−5) and *NFKB* signaling pathway (hsa04064 | hits = 12 out of 100 | FDR = 1.83e−4) to be the top deregulated pathways (Table S1). A total of 73 gene hits involved in pathways is depicted in the gene heat map (Figure S2). For the Toll-like receptor pathway, *CCL3L3, CCL4, CXCL8, IL1B, IRAK4, IRF7, MAP2K6, MYD88, NFKBIA, TLR1, TLR2, TLR4, and TLR5* genes were significantly enriched. As shown in Fig. 1, *MYD88, IRAK4*, and *NFKBIA* genes master a cascade of signaling transduction which leads to the expression of many inflammatory genes for cytokines, chemokines, endothelial adhesion molecules, and costimulatory molecules. *NFKBIA* gene was iterated in 9 out of the 10 top pathways, *MYD88* gene was enriched in 3 pathways, and *IRAK4* was the only gene located on chromosome X. Their diagnostic accuracy was compared to IL6, which is used in our hospital protocol as a gold standard test for inflammatory reaction in sepsis.

### Circulatory levels of Toll-like receptor signaling pathway genes in neonatal sepsis compared with healthy newborns

**Figure 2:**
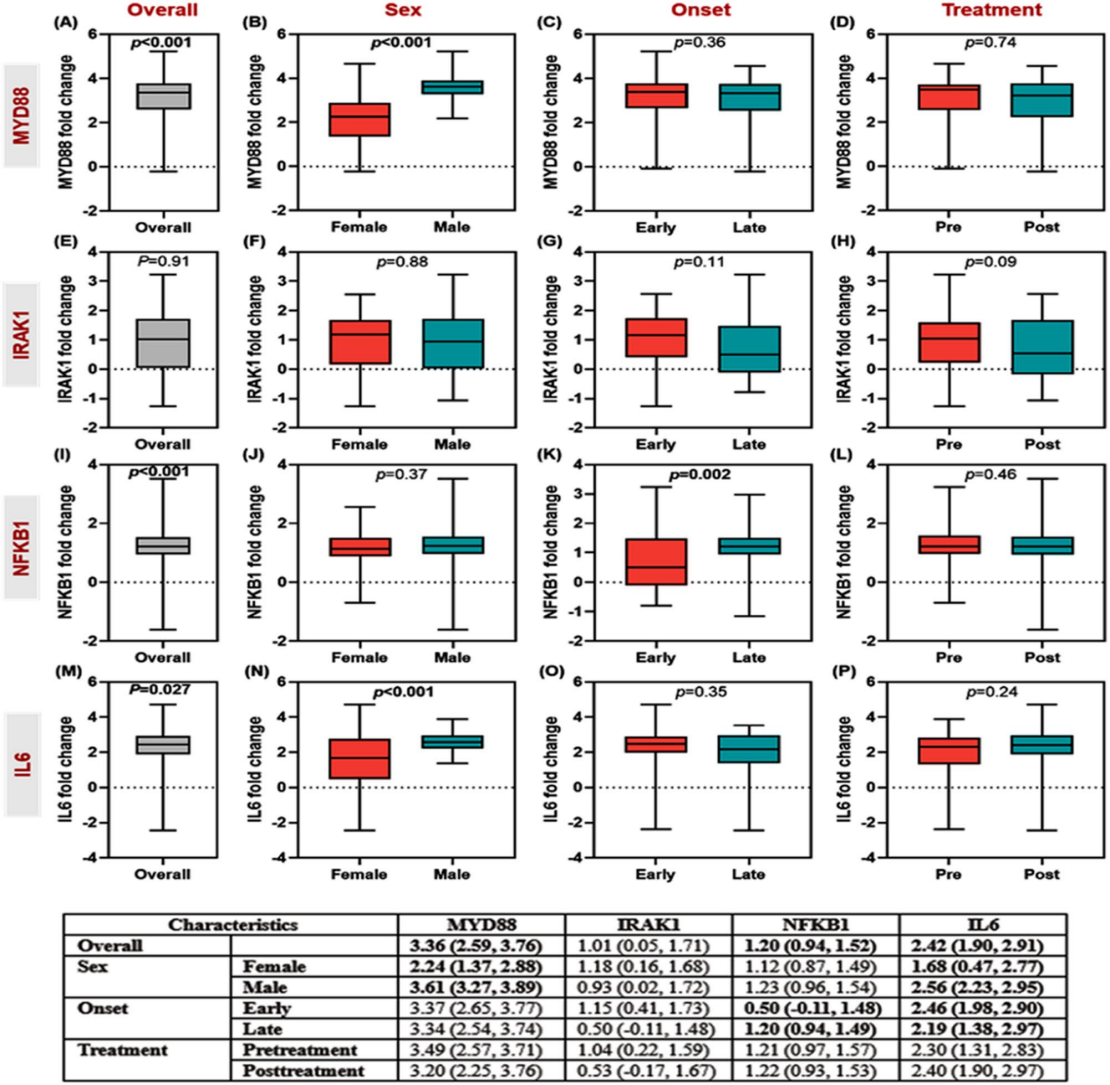
The relative expression level of circulatory Toll-like receptor signaling pathway genes. Four genes were analyzed: *MYD88*, *IRAK1*, *NFKB1*, and *IL6*. Whiskers and bars represented the median (Q1 and Q3). All values were log-transformed with the control level sets at the zero lines. Unpaired Mann–Whitney U test was used for all the analysis except the paired comparison between pretreatment and post-treatment, where Wilcoxon matched-pairs signed-rank test was employed instead. Bold *p*-values were significant at < 0.05. Transformed values of gene expression are presented as median (Q1 and Q3) in the attached table.

Fold changes of the four tested genes (*MYD88*, *IRAK1*, *NFKB1*, and *IL6*) in 81 males, and 43 females are shown in Fig. 2. Stratification analyses by sex and time of onset of the disease and comparing paired samples at admission with the second blood sample three days after treatment are shown. As demonstrated in Fig. 2, across all neonates, relative expression levels of *MYD88* (*p* < 0.001), *NFKB1* (*p* < 0.001), and *IL6* (*p* = 0.027) were significantly higher in sepsis cases compared to controls. Higher levels of MYD88 (*p* < 0.001) and IL6 (*p* < 0.001) were found in male infants compared to females. None of the gene levels were significantly altered after receiving treatment.

### Diagnostic accuracy of gene expression levels

ROC analysis revealed circulatory levels of *MYD88* and *NFKB1* transcripts to be good biomarkers for sepsis in neonates. The areas under the curves were 0.94 ± 0.02 (92.7 sensitivity, 87.3% specificity, *p* < 0.001) and 0.89 ± 0.02 (86.4% sensitivity, 75.4% specificity, *p* < 0.001), respectively. Regarding laboratory testing, both CRP and Immature/total WBCs ratio showed comparable diagnostic value, with AUC of 0.97 ± 0.01 (96.6% sensitivity, 76.5% specificity, *p* < 0.001) and 0.93 ± 0.03 (89.8% sensitivity, 82.4% specificity, *p* < 0.001) (Table 2).

**Table 2:** Receiver operator characteristic analysis for diagnostic accuracy testing of gene markers.

Test	Marker	AUC	SE	*P*-value	Sensitivity	Specificity
Laboratory data	WBCs	0.570	0.046	0.35	54.2%	58.8%
	I/T ratio	0.930	0.038	** < 0.001**	89.8%	82.4%
	CRP	0.973	0.013	** < 0.001**	96.6%	76.5%
Gene expression	*MYD88*	0.941	0.022	** < 0.001**	92.7%	87.3%
	*IRAK1*	0.517	0.046	0.82	57.6%	51.7%
	*NFKB1*	0.898	0.028	** < 0.001**	86.4%	75.4%
	*IL6*	0.674	0.043	**0.021**	69.5%	66.1%

Significant *P*-values are in bold. Abbreviations: WBC: white blood cells, I/T ratio: immature to total WBC ratio, CRP: C-reactive protein, AUC: area under the curve, SE: standard error.

### Clustering of patients into distinct phenotypes

**Figure 3:**
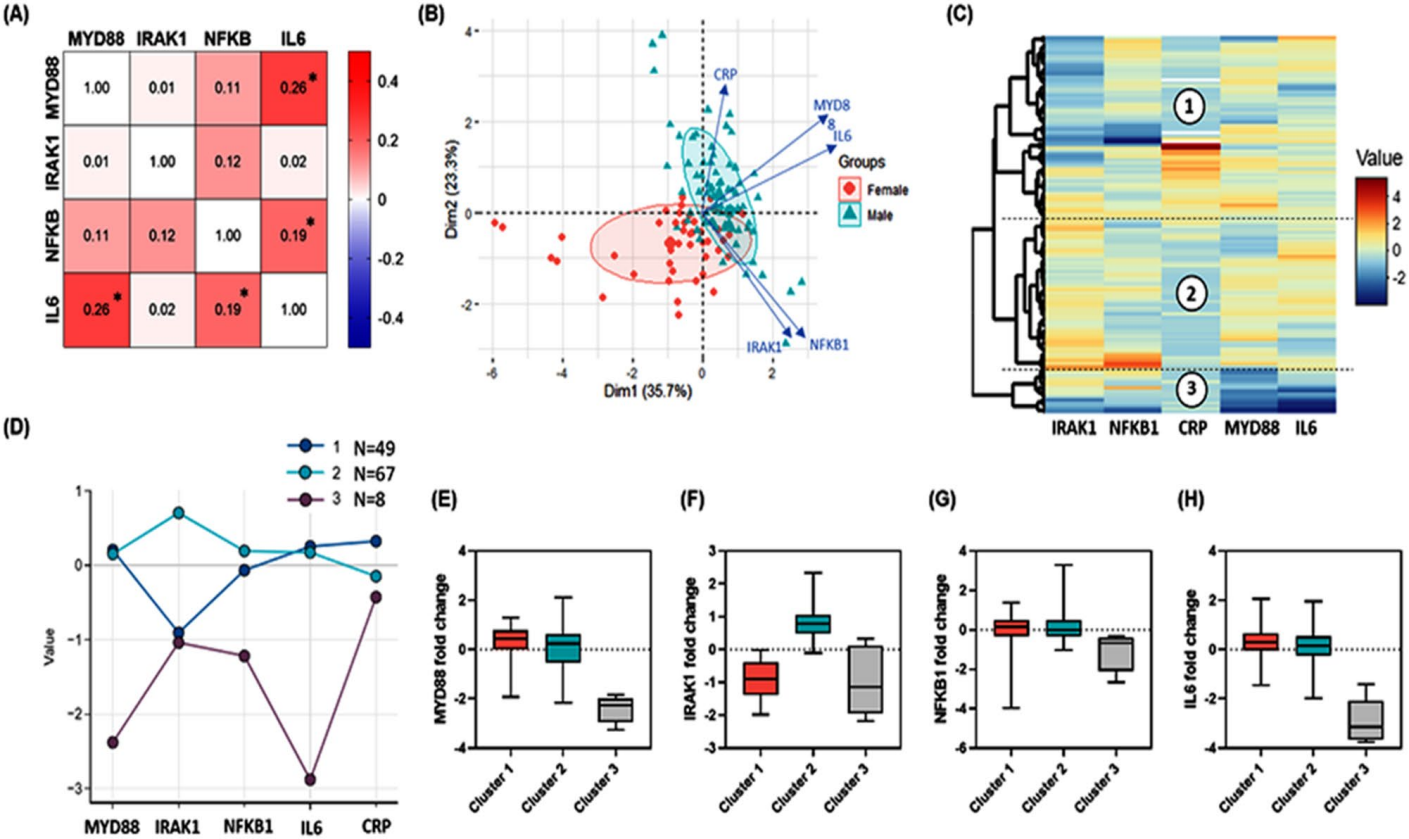
Clustering analysis. (**A**) Gene co-expression analysis for *MYD88*, *IRAK1*, *NFKB1*, and *IL6*. Spearman's correlation analysis was applied. Correlation coefficients are shown in corresponding cells. Asterisk sign is for significant correlations. Gene co-expression analysis across the 124 sepsis cohorts showed that *IL6* gene expression was directly correlated to *MYD88* (r = 0.26, *p* = 0.004) and *NFKB1* (r = 0.19, *p* = 0.039) gene levels. (**B**) Principal component analysis with axes 1 and 2 explaining variability in samples by 35.7% and 23.3%, respectively. The transcriptomic pattern showed clear demarcation between males and females with a small overlapping part. Over-expressed *MYD88* and *IL6* had a major contribution to the separation between male and female sepsis patients. (**C**) Hierarchical clustering analysis using the following parameters: Ward's minimum variance clustering method, Euclidian distance, scaling, and centering. The dendrogram shows the patients were clustered into three groups according to the pattern of 4 gene expression and C-reactive protein. (**D**) K-means clustering with scaling and centering. The five parameters used clustered patients into three groups: cluster 1 included 67 infants with low *IRAK1* gene expression, cluster 2 including 49 neonates characterized by high *IRAK1* gene expression, while cluster 3 of 8 patients exhibited remarkably lower *MYD88* and *IL6* expressions. (**E**–**H**) Box plots for the gene expression in each cluster.

Sepsis patients exhibited a different pattern of gene expression combinations. Gene co-expression analysis across the 124 sepsis cohorts showed that *IL6* gene expression was directly correlated to *MYD88* (r = 0.26, *p* = 0.004) and *NFKB1* (r = 0.19, *p* = 0.039) transcript levels (Fig. 3A). Clustering analysis split cohorts into three distinct groups according to their transcriptomic signature and CRP levels; cluster 1 included 67 infants with low *IRAK1* gene expression, cluster 2 including 49 neonates characterized by high *IRAK1* gene expression, while cluster 3 of 8 female patients exhibited remarkable lower expression of *MYD88* and *IL6* (Fig. 3B, C). This finding was confirmed by the PCA, where gene expression patterns showed good separation between males and females mainly by the high level of *MYD88* and *IL6* (Fig. 3D).

### Association of gene expression levels with clinical characteristics of the patients

As shown in Table 3, *MYD88* expression was upregulated in patients presented with necrotizing enterocolitis (*p* = 0.043), transient tachypnea (*p* = 0.043), poor feeding (*p* = 0.037), congenital pneumonia (*p* = 0.021), acquired pneumonia (*p* = 0.003), and jejunal atresia (*p* = 0.029). Elevated circulatory levels of the *MYD88* were associated with mortality (*p* = 0.007). *NFKB1* overexpression was associated late-onset sepsis (*p* = 0.002), cohorts with hemodynamic instability (*p* = 0.044), poor feeding (*p* = 0.032), and oliguria (*p* = 0.026). Moreover, *IL6* was significantly upregulated in males (*p* < 0.001), low-weight birth babies (*p* = 0.032), and infants of triple I mothers (*p* = 0.006). Cluster 1 was associated with temperature instability (*p* = 0.048) and poor feeding (*p* = 0.018). All cluster 3 were females, while males were more representative in cluster 1 (*p* < 0.001).

**Table 3:** Association of gene expression with clinical features.

Characteristics	*MYD88*	*IRAK1*	*NFKB1*	*IL6*	Clusters
Sex	** < 0.001**	0.88	0.37	** < 0.001**	** < 0.001**
Onset	0.65	0.07	**0.002**	0.35	0.44
Complications	0.06	0.95	0.47	0.45	0.45
Pulmonary hemorrhage	0.76	0.93	0.57	0.77	0.57
VAP	0.84	0.96	0.44	0.64	0.59
Air leak syndrome	0.79	0.80	0.84	0.92	0.75
Respiratory failure	0.58	0.41	0.39	0.60	0.49
Organ failure	0.13	0.90	0.27	0.12	0.71
Necrotizing enterocolitis	**0.043**	0.88	0.81	0.10	0.82
TTN	**0.043**	0.66	0.30	0.96	0.17
Temperature instability	0.79	0.05	0.67	0.71	**0.048**
Hemodynamic instability	0.13	0.81	**0.044**	0.43	0.45
Hypoxia	0.77	0.22	0.19	0.10	0.48
Lethargy	0.94	0.24	0.51	0.07	0.45
Hypoglycemia	0.13	0.09	0.60	0.94	0.21
Poor feeding	**0.037**	0.57	**0.032**	0.73	**0.018**
Seizures	0.17	0.28	0.41	0.80	0.42
Oliguria	0.62	0.74	**0.026**	0.92	0.37
RDS	0.62	0.87	0.63	0.53	0.68
Congenital pneumonia	**0.021**	0.89	0.48	0.72	0.78
Acquired pneumonia	**0.003**	0.12	0.48	0.60	0.07
Maternal antibiotic	0.81	0.51	0.52	0.23	0.47
Mode of delivery	0.23	0.25	0.19	0.93	0.88
Low birth weight	0.59	0.80	0.62	**0.032**	0.57
PROM	0.11	0.84	0.50	0.13	0.64
Maternal diabetes	0.13	0.16	0.51	0.65	0.22
Maternal anemia	0.29	0.91	0.30	0.30	0.88
Maternal hypertension	0.15	0.90	0.98	0.18	0.82
Pre-eclampsia	0.54	0.43	0.85	0.54	0.83
Triple I	0.42	0.63	0.11	**0.006**	0.24
Preterm	0.94	0.53	0.41	0.69	0.9
IUGR	0.89	0.85	0.58	0.16	0.74
Total parental nutrition	0.65	0.50	0.80	0.38	0.17
Umbilical venous catheter	0.82	0.73	0.98	0.13	0.72
Congenital heart disease	0.45	0.61	0.65	0.29	0.59
Jejunal atresia	**0.029**	0.75	0.68	0.28	0.85
Inguinal hernia	0.40	0.79	0.59	0.44	0.58
Positive culture	0.98	0.68	0.82	0.68	0.38
Type of organism	0.24	0.16	0.65	0.57	0.20
Mechanical Ventilation	0.54	0.31	0.46	0.30	0.14
Surfactant therapy	0.17	0.16	0.27	0.18	0.12

*P*-values are shown in the table. Mann–Whitney U, Kruskal–Wallis tests were used for association with genes, while a two-sided Chi-square test was performed for association with the three clusters. Significant *P*-values are in bold. Abbreviations: Triple I: intrauterine inflammation, infection, or both (a new concept of chorioamnionitis), IUGR: intrauterine growth retardation. LBWB: low birth-weight baby. PROM: premature rupture of membrane. TTN: transient tachypnoea of newborns, VAP: ventilation-association pneumonia, RDS: respiratory distress syndrome.

Spearman's correlation analysis of the gestational age with gene expression of the studied four genes revealed a very weak correlation coefficient ranging from 0.02 to 0.08 (Figure S3).

In those neonates with positive blood culture, cohorts were categorized into gram-positive and gram-negative groups. Gene expression analysis revealed no significant difference in both groups (Figure S4). In addition, the type of organism in positive blood culture patients did not show a significant impact on mortality risk as illustrated in Cox proportionate regression model (univariate analysis: hazard's ratio = 0.61, 95%CI = 0.22–1.64, *p* = 0.33, and multivariate analysis: HR = 0.36, 95%CI = 0.12–1.11, *p* = 0.08) (data not shown).

### Association of gene expression levels with survival

**Figure 4:**
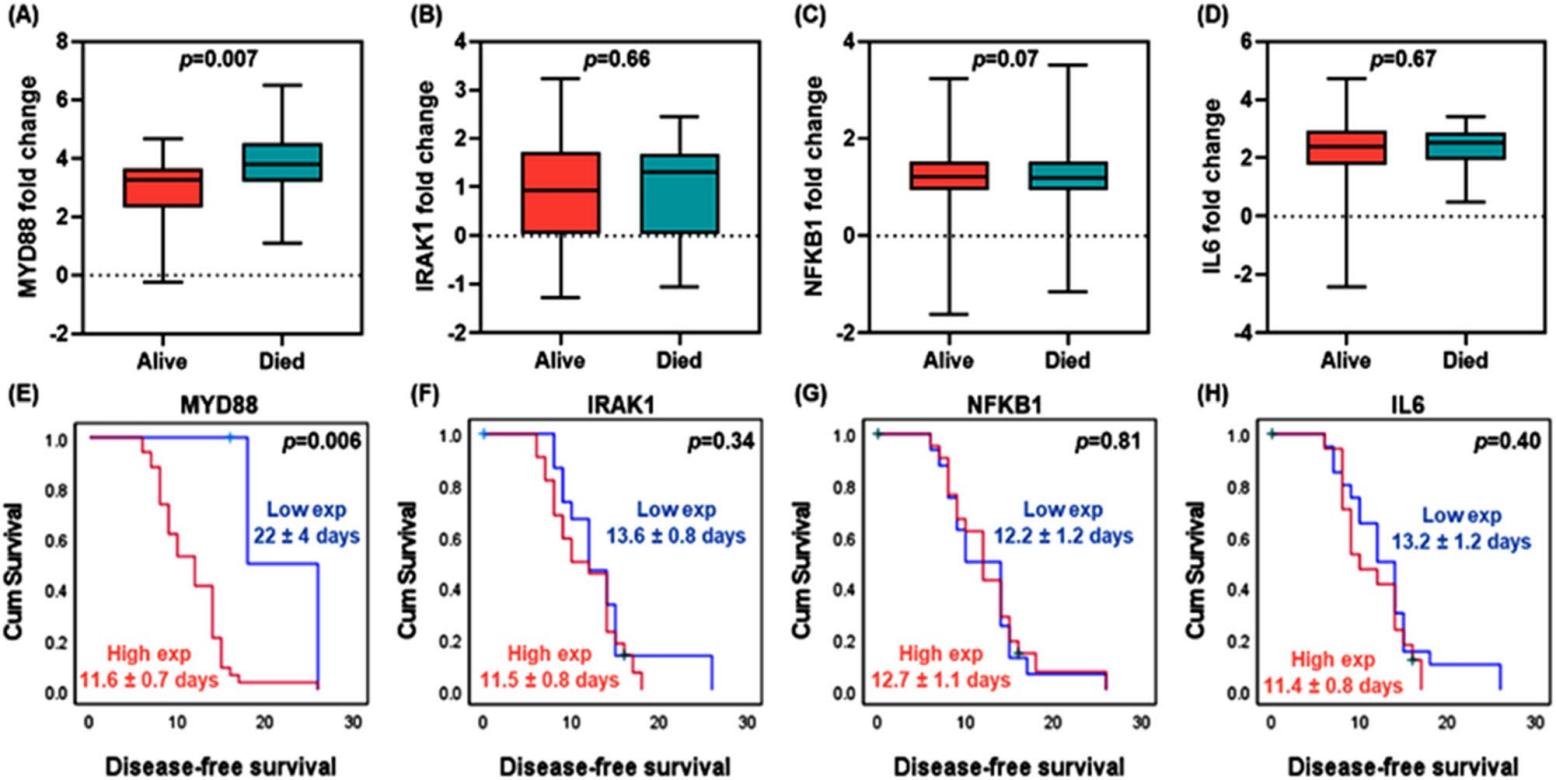
Survival analysis in sepsis patients. (**A**–**D**) Box plots for the expression level of alive and expired neonates. Mann–Whitney U test was used. (**E**–**H**) Kaplan–Meier survival curves. Log-Rank test was used to test the significant difference.

In the current study, gene expression analysis in 88 sepsis cohorts who survived, and 36 deceased neonates revealed a significant upregulation of *MYD88* in expired cases (median = 3.81, IQR = 3.2–4.5) compared to alive ones (median = 3.27, IQR = 2.3–3.6), *p* = 0.007. Kaplan–Meier curves showed that patients with high levels of circulatory *MYD88* gene were associated with poor survival (11.6 ± 0.7 days versus 22.0 ± 4 days, *p* = 0.006) (Fig. 4).

### Predictor risk factors for survival

**Figure 5:**
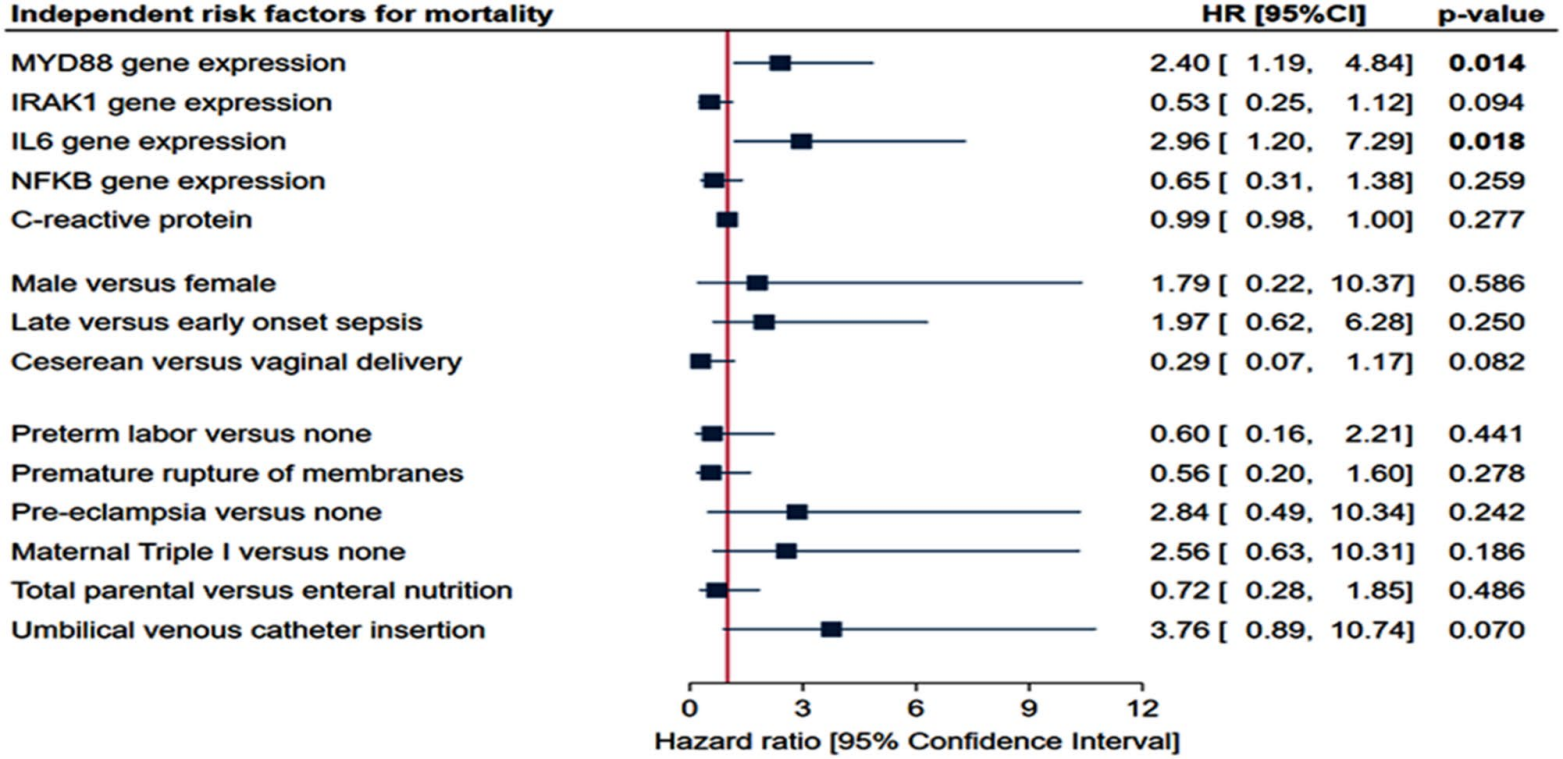
Multivariate analysis for risk factors of mortality. Cox hazard proportionate regression analysis was employed. Hazard ratio (HR) and confidence intervals (CI) are shown with p values set significant at < 0.05.

Multivariate analysis included gene expression, demographic data, and risk factors for sepsis. Patients with high circulatory levels of MYD88 (HR = 2.4, 95% CI = 1.19–4.84, *p* = 0.014) and IL6 (HR = 2.96, 95%CI = 1.20–7.29, p = 0.018) transcripts had 2.5 times more risk of mortality by Cox regression analysis (Fig. 5).

### Functional enrichment analysis of Toll-like receptor signaling pathway genes

**Figure 6:**
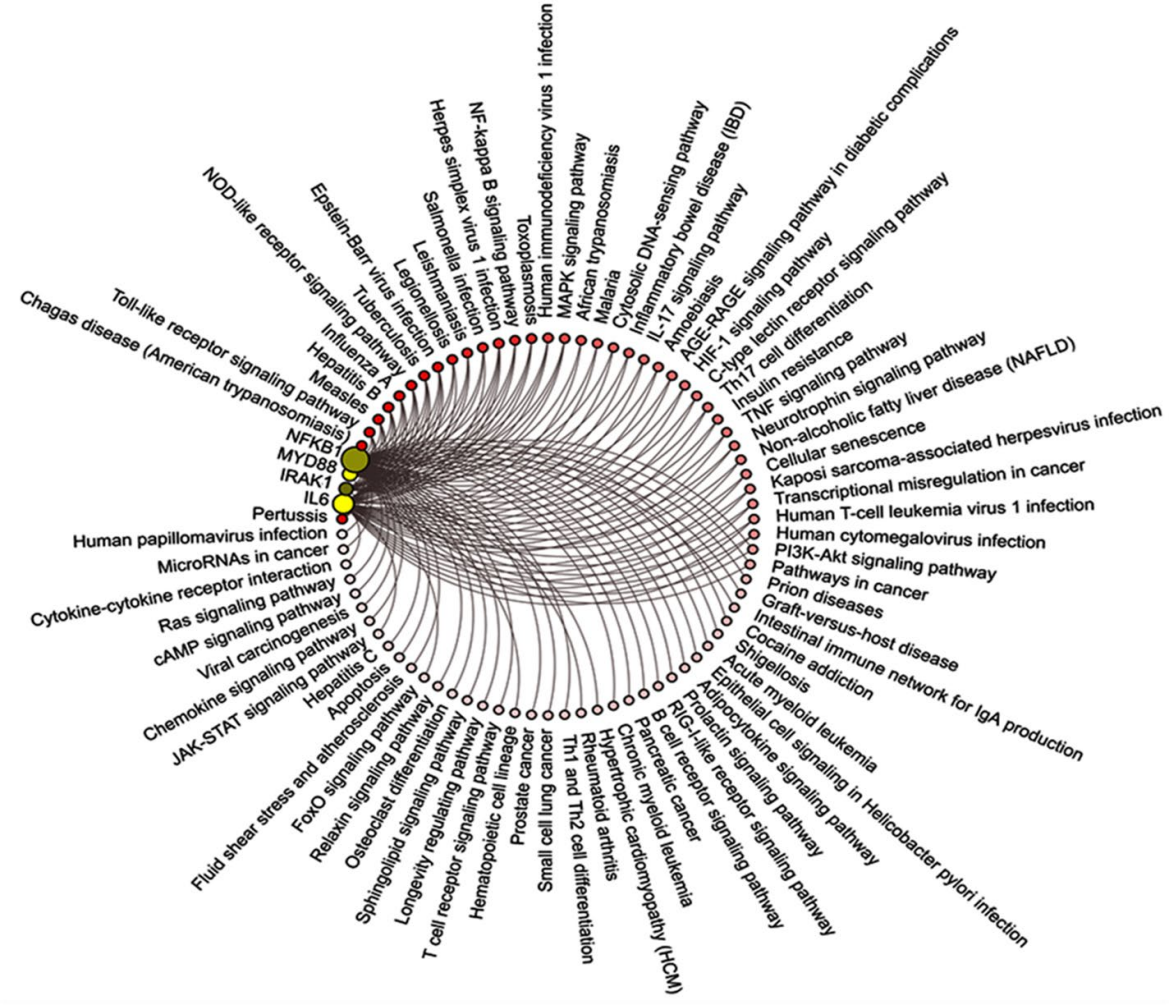
Pathway enrichment of *MYD88*, *IRAK1*, *NFKB1*, and *IL6* genes. The query genes are displayed as nodes colored by their abundance, with yellow corresponding to high abundance. Enriched pathways and diseases are colored according to the enrichment test *P*-value from "Enricher", with darker red corresponding to more significant enrichment. Edges connect enriched pathways/diseases and their members in the query gene set [Data source: pinet-server.org].

Pathway analysis for the four studied genes showed them to be involved in multiple inflammatory-related pathways and host immune responses (Fig. 6).

## Discussion

Although the sepsis incidence continues to increase and impacts a wide range of ages, our understanding of human response to sepsis across different age groups remains inadequate and limits our ability to modify outcomes^6^. Given the essential roles TLR and NFKB-pathways play in sepsis as identified previously^11,12^ and through our in silico analysis, this work aimed to explore the transcriptomic signature and the clinical utility of *MYD88, IRAK1, NFKB*, and *IL6* in a sample of neonatal sepsis and to correlate the gene signature with the clinic-laboratory data.

In this study, significant upregulation of *MYD88* was identified in neonates with sepsis relative to uninfected controls. Interestingly, *MYD88* mRNA showed a sex-specific signature being higher in male neonates than females, contributing to the clustering of both groups by the principal component analysis. The findings also highlight the potential clinical utility of this transcript as a diagnostic/prognostic molecular biomarker in terms of having a high area under the ROC curve and associating with unfavorable clinical phenotypes (e.g. necrotizing enterocolitis, transient tachypnea, poor feeding, congenital and acquired pneumonia, jejunal atresia) and poor survival.

Given the central role MyD88 plays in TLR signaling, TLR-induced death, and the innate immune response activation^13^, among others (as shown in Fig. 6), it is not surprising to find *MYD88* overexpression in PBMC of the sepsis cohort in line with the findings of previous clinical studies and experimental septic models^13–16^. For example, Salomão et al. show upregulation of all TLR-signaling pathway-related genes in neutrophils of septic patients, which were persisted across the different stages of sepsis^13,14^. Adib-Conquya et al. also reported a significant upregulation of MyD88s in monocytes of septic patients compared to either resuscitated patients after cardiac arrest or healthy controls. Their transfection experiments identified that the short form of "MyD88" could negatively regulate "TLR2-dependent NF-B response"^15^. Furthermore, Khailova et al. confirmed the significant increase of MyD88 with other assessed TLR pathway-related molecules (TLR-2 and NFΚ) in the lungs of septic mice model of cecal ligation and puncture peritonitis relative to healthy shams^16^. This overexpression was ameliorated on "*Lactobacillus rhamnosus* GG" or "*Bifidobacterium longum*" probiotic treatment.

The present finding of *MYD88* upregulation showed sex disparity with significantly higher levels in male neonates than females, contributing to the previous observations in which females exhibited a less exaggerated immune response than males^17,18^. While the exact mechanism underlying this observation is not conclusive, hormonal and epigenetic factors were proposed to contribute to this phenomenon by impacting the pathogen-specific inflammatory responses (particularly lipopolysaccharides) and the immunological differences^18–20^. Naugler et al. have found that "MYD88-dependent activation of IL6 production" could be negatively regulated by estrogen in their model of hepatocellular carcinoma induced by liver inflammation^21^. Estrogen has displayed a suppressive effect on inflammation, mainly via downregulating the NFKB1 transcriptional activity with a subsequent decrease in pro-inflammatory cytokine/chemokine production, including IL6^22–24^. El Sabeh et al., in their recent preprint, revealed that MYD88/estrogen receptor-α interaction during the inflammatory signaling might contribute to the gender-dependent bias in the inflammatory response^25^. In addition, Crisostomo et al. identified that the stimulated female-derived murine mesenchymal stem cells have demonstrated less inflammatory response, including IL6 and tumor necrosis factor-α production, compared with the male-derived ones^26^. All these observations, including ours, highlight the importance of running "sex-based therapeutic interventions," which will improve neonatal outcomes, particularly in a neonatal intensive care unit (NICU)^27^.

Signals transduced by MYD88 should continue to the nucleus to upregulate the pro-inflammatory gene expressions, including the *IL6* gene, through the transcriptional factor NFKB (shown in Fig. 1), which orchestrates the immune response through multiple downstream targets leading to inflammatory reactions and ultimately severe phenotype^28^. This supports in part the observed upregulation of *IL6* and *NFKB1* in septic neonates and association with some features of poor outcomes, including low-weight birth babies and infants of triple I mothers (in case of IL6), and hemodynamic instability, poor feeding, and oliguria (in case of *NFKB1*).

The pro-inflammatory cytokine IL6 is expressed by immune cells (macrophages, dendritic cells, B cells, and epithelial cells). It has been found to "exhibit a more prolonged response to the pathogenic challenge", even in the period of temperature stabilization, in comparison to other cytokines^20^. Such type of pleiotropic interleukin mediates its molecular signaling through JAK/STAT and MAPK pathways and is involved in many biological processes, including cell survival/apoptosis, T cell maturation, T helper-1/2/17 differentiation, and inflammation^29^. It showed higher levels in neonatal males versus females in the present study and adult males with sepsis versus females in earlier studies^30–32^ and after administering lipopolysaccharides in experimental work^19^. High serum levels of IL6 showed a correlation with the low circulating lymphocyte counts and correlated with a high risk of developing acute respiratory distress syndrome^33^. These findings could support the predictive role that such cytokine plays in septic episode severity as proposed by Wang and colleagues^34^ and highlighting the IL6 as a promising target for the current monoclonal antibody therapy in the clinics^35^.

Although recent work suggests that a different gene expression signature might be present in the case of different bacteria (gram-positive *vs.* gram-negative)^36^, we found no significant difference between neonates with positive culture results of gram-positive and gram-negative. Also, despite some in vitro stimulation experiments (to imitate sepsis-like conditions), revealed that conventional antibiotic therapy might be associated with immunomodulatory properties associated with the change in transcriptomic signature for some TLRs and related cytokines^37,38^, none of the studied gene transcript levels in the present study were significantly altered at the second sampling time point (after three days of receiving antibiotics). It is worth noting that what was found in in vivo and in vitro studies does not always translate to the clinic. Also, it does not discount the possibility that the gene signatures could be normalized with a longer treatment time. Furthermore, differences in the study design, mammalian cells investigated, bacterial species, class of antibiotics tested, and sepsis stage, among others, should be considered.

This study is limited by the relatively small sample size and the study design (a case–control study), hence large-scale and follow-up studies are recommended. Also, it was difficult to find healthy preterm neonates during sample collection in our hospital and obtain parental consent for sample withdraw in such cases (if any); hence, this study included full-term neonates as a control group. This issue (matched case–control groups regards the gestational age) should be considered in future studies. Furthermore, because of the small volume of blood collected in this work, the authors could only investigate the expression of the studied genes at the level of mRNA. It would be useful to demonstrate the protein levels of the studied genes in future studies as the mRNA levels may not always reflect the corresponding protein expression due to the posttranscriptional processing. Indeed, the role of other genetic/epigenetic and environmental factors should be considered.

In light of the present findings, the development and validation of new approaches for neonatal sepsis treatment are recommended, such as the potential use of TLR-pathway antagonists/inhibitors to limit the TLRs association with MyD88 preferentially and reduces the NFKB1 activity and/or IL6^8,39–41^. Furthermore, as mentioned earlier, clinically relevant sex-based therapeutic strategies should be implicated as adjunct approaches to the ordinary ones to increase the potentially favorable outcomes in male neonates.

## Materials and methods

### Study subjects

This prospective case–control descriptive study enrolled 124 consecutive neonates with suspected or confirmed sepsis and 17 healthy neonates as controls recruited from the NICU of SCU hospitals from December 2018 to November 2019. The patients were enrolled according to the inclusion/exclusion criteria with laboratory evidence. The neonates with gestational age from 28–40 weeks confirmed by the new Ballard score^42^ and expected to be diagnosed clinically as neonatal sepsis were included. Neonates with a gestational age below 28 weeks, a history of perinatal hypoxia, hypoxic-ischemic encephalopathy, or gross congenital anomalies and genetic syndromes were excluded. If mothers had positive hepatitis C or B infection or were known to have a history of misuse drug intake during pregnancy, the neonates were also excluded. The controls included 17 samples of healthy full-term neonates collected in the same period during the routine screening of serum bilirubin. Because of the difficulty of blood sampling in some neonates, the drop-out sample (due to non-survivors), and the mother's non-approval for including a second sampling of their neonates, only 43 paired samples were available for the comparison.

### Clinical assessment

Each participant was subjected to history taking from the mother to detect any sign of sepsis, full maternal history, including maternal age, gravidity, and parity, medical history, details of labor with an emphasis on any prenatal hazards (as pre-eclampsia, premature rupture of membranes, antepartum hemorrhage, or intrapartum fever), detailed perinatal history of neonates, including gestational age, mode of delivery, early postnatal cyanosis, jaundice or convulsions, and full birth record, including methods and duration of resuscitation and mode of lactation (if present). The clinical data included (1) gestational age assessment, weight, and sex of the full-term neonates, (2) general and systemic examination, including (a) the respiratory system: tachypnea, apnea, increased ventilator support, and oxygen desaturation, (b) the cardiovascular system: bradycardia, pallor, hypotension, and decreased perfusion (c) metabolic changes: hypothermia, hyperthermia, feeding intolerance, glucose instability, metabolic acidosis, and (d) neurologic changes: lethargy, hypotonia, and decreased activity.

### Sample collection

Five milliliters of peripheral blood were withdrawn on admission for 124 neonates under aseptic conditions for routine chemistry and immunological assessment (2 ml on plain tubes), complete blood count (1 ml on EDTA tubes), blood culture (1 ml), and genetic analysis (1 ml on EDTA tubes). The latter test tubes were transferred immediately to the genetic lab within 20 min to be centrifuged with separation of the buffy coat in sterile Eppendorf for the subsequent genetic analysis. Sampling was then repeated after three days of treatment (as post-treatment samples) for the available 43 neonates.

### Blood culture

The collected venous blood was inoculated directly into blood culture medium vials and send to the clinical microbiology laboratory for cultivation. In brief, the blood cultures were incubated aerobically at 37 °C and observed daily for the first three days to identify any visible microbial growth. Simultaneously, subcultures were made during three consecutive days on enriched and selective media, including chocolate, MacConkey, blood, and mannitol salt agar plates. The subcultures were examined for growth after incubation for 24–48 h. The protocol was repeated until the 7th day before blood culture was considered sterile^43^.

### In silico selection of candidate pathway

Gene Expression Omnibus (GEO) database, a public functional genomics data repository, was screened for RNAseq experiments on neonatal sepsis. Transcriptomic signature of GSE138712 experiment was utilized for analysis, for which whole blood specimens of nine preterm neonates (< 30 weeks gestational age) with late-onset sepsis were compared to another nine age-matched preterm infants without sepsis. Data was downloaded from GEO RNA-seq Experiments Interactive Navigator (GREIN (ilincs.org). Transcriptomic analysis of the GEO dataset was performed using NetworkAnalyst (www.networkanalyst.ca). Differentially expressed genes (DEGs) were identified using Limma R package (false discovery rate “FDR” < 0.05 and |FC|> 1.0). Pathway enrichment analysis was performed in String database version 11.0 (string-db.org).

### Gene signature analysis

#### RNA extraction and reverse transcription

Extraction of total RNA from peripheral blood mononuclear (PBMN) cells was done using a Qiagen RNeasy RNA extraction kit (Cat No. 74106), following the manufacturer's protocol. All samples were treated with RNase-free DNase I (Qiagen, Hilden, Germany) for 2 h at 37 °C. RNA concentration and purity at A260:A280 ratios were determined by NanoDrop ND-1000 spectrophotometer (NanoDrop Tech., Inc. Wilmington, DE, USA) followed by agarose gel electrophoresis check for RNA integrity. For reverse transcription (RT) reaction, High-Capacity cDNA Reverse Transcription Kit (P/N 4368814, Thermo Fisher Scientific, Waltham, MA, USA) was used. Extracted RNA (10 ng) was added to "2 × RT reaction mix (10 µL) containing 10 × RT Buffer (2 µL), 25 × dNTP Mix (100 mM; 0.8 µL), 10 × RT random primers (2 µL), MultiScribe™ Reverse Transcriptase enzyme (1 µL), RNase inhibitor (1 µL), and nuclease-free water (3.2 µL)", with the application of appropriate negative controls in each run, The PCR reaction subjected to 25 °C for 10 min, followed by 37 °C for 120 min, and finally 85 °C for 5 min, then hold at 4 °C in a Master cycler Gradient Thermocycler (Eppendorf, Hamburg, Germany)^44^.

#### Quantitative reverse transcriptase-polymerase chain reaction

The study four genes relative expressions were quantified using TaqMan® assays (Applied Biosystems, assay ID Hs01573837_g1 for *MYD88*, Hs00155570_m1 for *IRAK1*, Hs00765730_m1 for *NFKB1*, and Hs00174131_m1 for *IL6*), glyceraldehyde-3-phosphate dehydrogenase (*GAPDH)* endogenous control assay (ID: Hs03929097_g1), and Universal PCR master mix II, No UNG (2 ×) (TaqMan, Applied Biosystems) on StepOne™ Real-Time PCR System (Applied Biosystems). All reactions were run in duplicate, and a "No-template" and a "No-RT" controls were included in each run. Each 96-well plate run initially at 95 °C for 5 min, followed by 40 cycles of denaturation at 95 °C (15 s), annealing at 60 °C (1 min), and elongation at 72 °C (1 min). Real-time PCR was run following the "Minimum Information for Publication of Quantitative Real-Time PCR Experiments (MIQE) guidelines"^45^. The fold change of the transcriptomic signature of the four genes in the neonates with sepsis relative to the mean value of the controls was calculated using the LIVAK method based on the quantitative cycle (Cq) values with the equation (2^−ΔΔC*q*^)^46^.

### Statistical analysis

Data analysis was performed using SPSS version 27.0, GraphPad Prism version, and RStudio 1.3.1056. Using the G*Power 3.1.9.2. with the specified study design (gene expression), alpha error = 0.05, an effect size = 0.74, and a total sample size of 138 can give 81% power of the study. http://www.gpower.hhu.de/A two-sided Chi-Square test was employed for categorical variables, and Mann–Whitney or Kruskal Wallis was applied for quantitative data. Univariate logistic regression analysis was performed, and results were reported as odds ratio (OR) and 95% Confidence intervals (CI). Spearman's correlation analysis was used. The statistical significance cutoff level was set at *P* value < 0.05. Hierarchical clustering analysis and K-means clustering were generated and visualized using BioVinci (Bioturing, San Diego, CA, USA) through the following parameters: Ward's minimum variance clustering method, Euclidian distance, dendrogram, scaling and centering. Gene expression was categorized at the median cutoff values into high and low expression. To compare the survival in high and low expressor groups, "Kaplan–Meier" curves were plotted. Cox Hazards Proportional Regression analysis was carried out to identify independent risk factors for mortality, and results are reported as hazards ratio (HR) and 95%CI. Multivariate analysis was executed for data exploration in the principal component analysis (PCA) using "Psych, Factoextra, FactoMineR, ggplot2, ggpubr, and magrittr" packages.

### Institutional review board statement

The study was conducted according to the Declaration of Helsinki's guidelines and approved by the Ethics Committee of Suez Canal University, Faculty of Medicine, Ismailia, Egypt (Approval No. 3660).

### Author's agreement of originality and statement of copyright transfer

This is an original article that has not been published in any other publication.

### Informed consent

Informed consent was obtained from all included neonate caring relatives in the study.

## Supplementary Information

Supplementary Information.

## Data Availability

All data generated or analyzed during this study are included in this submitted article and Supplementary Materials.
