# Supplementary material for: MYD88, NFKB1, and IL6 transcripts overexpression are associated with poor outcomes and short survival in neonatal sepsis
Source: Sci Rep. 2021 Jun 28;11:13374. doi: 10.1038/s41598-021-92912-7 (PMC8238937; doi:10.1038/s41598-021-92912-7)
Supplement: Supplementary file 1 — Supplementary Information. [file 41598_2021_92912_MOESM1_ESM.pdf]

Research Article

MYD88, NFKB1, and IL6 transcripts overexpression are associated with poor outcomes and short survival in neonatal sepsis

Nouran B AbdAllah[1], Eman A Toraih[2,3], Essam Al Ageeli[4], Hala Elhagrasy[1], Nawal S Gouda[5,6], Manal S Fawzy[7,8*], Ghada M Helal[9]

[1] *Department of Pediatrics, Faculty of Medicine, Suez Canal University, Ismailia, Egypt;* *nouranb.abdallah@gmail.com*

[2] *Department of Surgery, Tulane University, School of Medicine, New Orleans, Louisiana, USA;* *etoraih@tulane.edu*

[3] *Genetics Unit, Department of Histology and Cell Biology, Faculty of Medicine, Suez Canal University, Ismailia, Egypt;* *emantoraih@gmail.com*

[4] *Department of Clinical Biochemistry (Medical Genetics), Faculty of Medicine, Jazan University, Jazan, Saudi Arabia;* *dr.ageeli@gmail.com*

[5] *Department of Medical Microbiology and Immunology, Faculty of Medicine, Mansoura University, Mansoura, Egypt;* *nawalsalama@gmail.com*

[6] *Department of Microbiology, Faculty of Medicine, Northern Border University, Arar, Saudi Arabia*

[7] *Department of Medical Biochemistry and Molecular Biology, Faculty of Medicine, Suez Canal University, Ismailia, Egypt;* *manal_mohamed@med.suez.edu.eg*

[8] *Department of Biochemistry, Faculty of Medicine, Northern Border University, Arar, Saudi Arabia*

[9] *Department of Medical Biochemistry, Faculty of Medicine, Mansoura University, Mansoura, Egypt;* *ghadahelal76@yahoo.com*

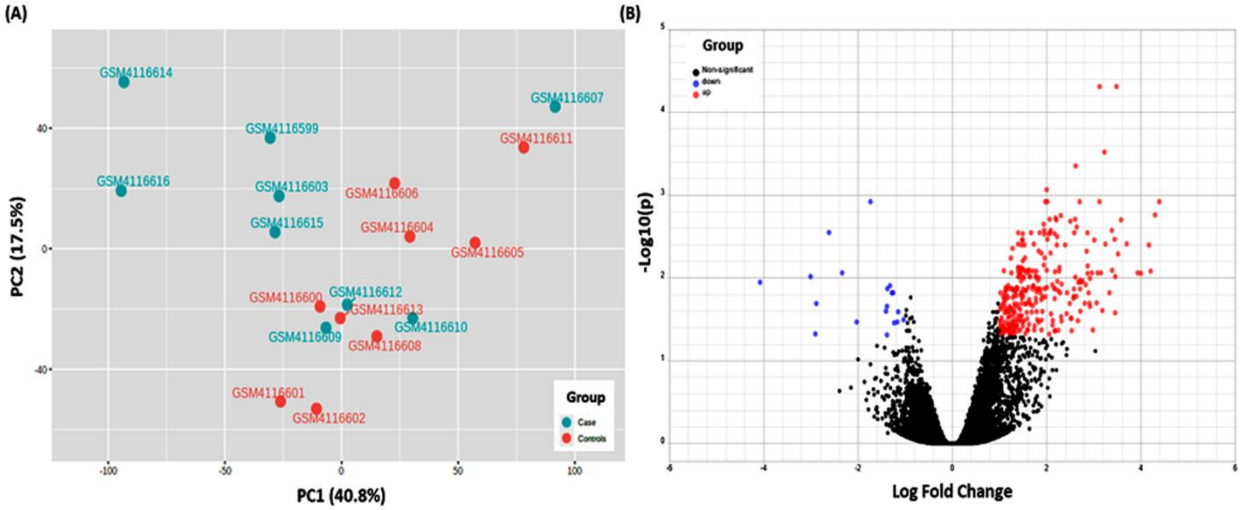

Figure S1. Transcriptomic analysis of GSE138712 RNAseq experiment of neonatal sepsis. Transcriptomic signature of whole blood specimens of 18 preterm infants (nine with sepsis and nine without sepsis) was compared. (A) Principal component analysis showing incomplete demarcation between cases and controls. (B) Volcano plot showing the 324 differentially expressed genes. Each dot represents a gene: upregulated in red and downregulated in blue, with insignificant gene expression in black. Genes were filtered by having absolute fold change over 1.0 and adjusted p-value <0.05.

Table S1. Pathway enrichment analysis

**Pathway**	**Total**	**Expected**	**Hits**	***p*-Value**	**FDR**
Toll-like receptor signaling pathway	104	2.2	13	2.49E-07	7.93E-05
NF-kappa B signaling pathway	100	2.12	12	1.15E-06	0.000183
TNF signaling pathway	110	2.33	12	3.21E-06	0.00034
Osteoclast differentiation	128	2.71	12	1.56E-05	0.00114
NOD-like receptor signaling pathway	178	3.77	14	2.27E-05	0.00121
IL-17 signaling pathway	93	1.97	9	0.000145	0.0066
Cytosolic DNA-sensing pathway	63	1.34	7	0.000345	0.0122
Staphylococcus aureus infection	68	1.44	7	0.000553	0.016
RIG-I-like receptor signaling pathway	70	1.48	7	0.00066	0.0175
Th17 cell differentiation	107	2.27	8	0.00189	0.0401

FDR: false discovery rate, NF: nuclear factor, NOD: nucleotide-binding oligomerization domain, IL-17: Interleukin-1, RIG-I: retinoic acid-inducible gene-I-, Th17: T helper cells. Data source: STRING version 11.0.

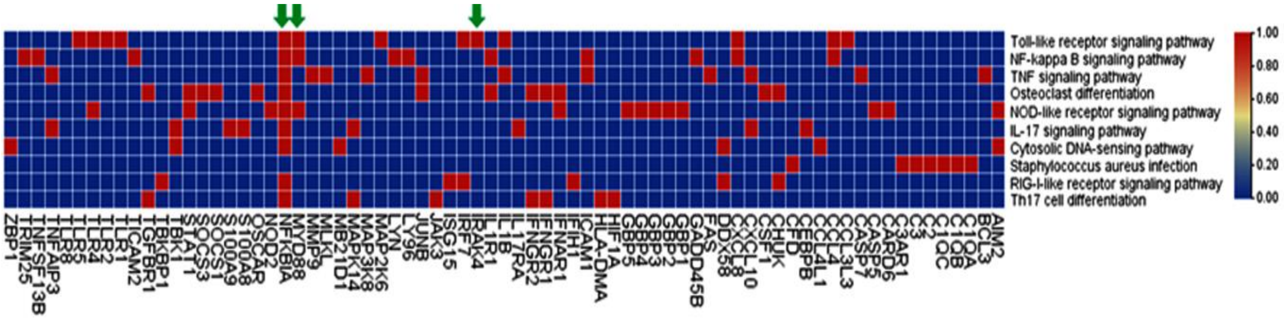

Figure S2. Gene heatmap of deregulated genes enriched in top activated or inhibited pathways in neonatal sepsis. Data from the GSE138712 RNAseq experiment was used for analysis. Heatmap involved analysis of 73 genes. The red box indicates a positive hit, the blue box indicates not significantly involved in the pathway. The colored sidebar indicates the level of *P*-value significance. The arrows indicated the selected genes in the present study.

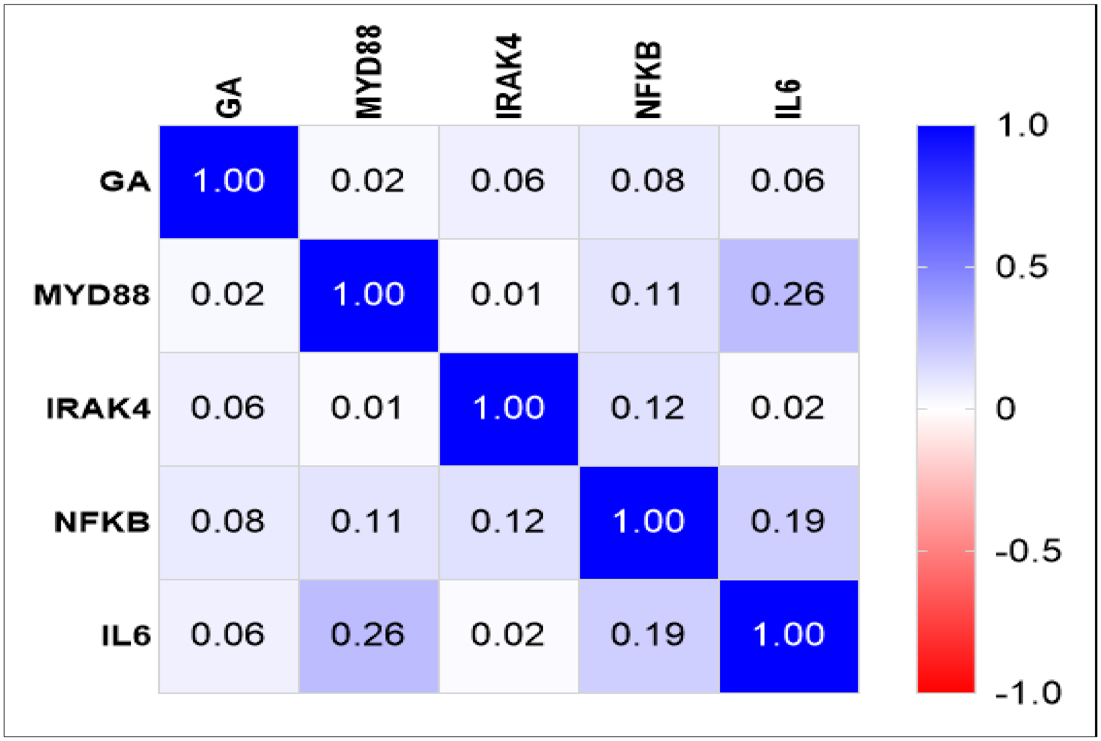

Figure S3. Correlation analysis between the gestational age (GA) and the studied genes in sepsis patients. Spearman's correlation analysis was applied. The sidebar indicates the level of significance.

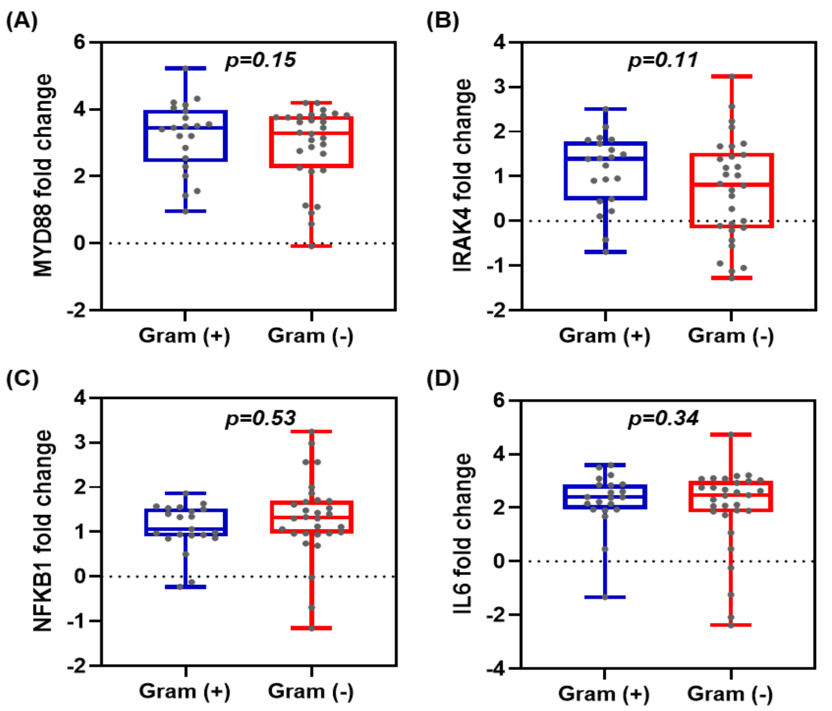

Figure S4. Comparison between gram-positive and gram-negative cohorts. Box plots for the expression level of gram-positive and negative blood culture tests in neonates. Y-axis represents the logarithmic transformation of gene expression, and X-axis represents the groups. Mann-Whitney U test was used.
